# Xylobiose, an Alternative Sweetener, Ameliorates Diabetes-Related Metabolic Changes by Regulating Hepatic Lipogenesis and miR-122a/33a in db/db Mice

**DOI:** 10.3390/nu8120791

**Published:** 2016-12-05

**Authors:** Eunjin Lim, Ji Ye Lim, Eunju Kim, Yoo-Sun Kim, Jae-Ho Shin, Pu Reum Seok, Sangwon Jung, Sang-Ho Yoo, Yuri Kim

**Affiliations:** 1 Department of Nutritional Science and Food Management, Ewha Womans University, Seoul 03760, Korea; leemeunjin@daum.net (E.L.); godlovesme86@hanmail.net (J.Y.L.); eunju831@naver.com (E.K.); tidygirlss@naver.com (Y.-S.K.); 2 Department of Biomedical Laboratory Science, Eulji University, Seongnam-si, Gyunggi-do 13135, Korea; shinjh@eulji.ac.kr (J.-H.S.); seok@eulji.ac.kr (P.R.S.); 3 R&D Center, TS Corporation, Incheon 22300, Korea; chemjsw@ts.co.kr; 4 Department of Food Science & Biotechnology, and Carbohydrate Bioproduct Research Center, Sejong University, Seoul 05006, Korea; shyoo@sejong.ac.kr

**Keywords:** xylobiose, lipogenesis, db/db mice, microRNA, inflammation

## Abstract

Type 2 diabetes is a major public health concern worldwide. Xylobiose (XB) consists of two molecules of d-xylose and is a major disaccharide in xylooligosaccharides that are used as prebiotics. We hypothesized that XB could regulate diabetes-related metabolic and genetic changes via microRNA expression in db/db mice. For six weeks, C57BL/KsJ-db/db mice received 5% XB as part of the total sucrose content of their diet. XB supplementation improved glucose tolerance with reduced levels of OGTT AUC, fasting blood glucose, HbA1c, insulin, and HOMA-IR. Furthermore, XB supplementation decreased the levels of total triglycerides, total cholesterol, and LDL-C. The expression levels of miR-122a and miR-33a were higher and lower in the XB group, respectively. In the liver, expressions of the lipogenic genes, including, fatty acid synthase (FAS), peroxisome proliferator activated receptor γ (PPARγ), sterol regulatory element-binding protein-1C (SREBP-1C), sterol regulatory element-binding protein-2 (SREBP-2), acetyl-CoA carboxylase (ACC), HMG-CoA reductase (HMGCR), ATP-binding cassette transporter G5/G8 (ABCG5/8), cholesterol 7 alpha-hydroxylase (CYP7A1), and sterol 12-alpha-hydroxylase (CYP8B1), as well as oxidative stress markers, including superoxide dismutase 1 (SOD1), superoxide dismutase 2 (SOD2), glutathione peroxidase (GPX), and catalase, were also regulated by XB supplementation. XB supplementation inhibited the mRNA expressions levels of the pro-inflammatory cytokines, tumor necrosis factor (TNF)-α, interleukin (IL)-1β, interleukin (IL)-6, and monocyte chemoattractant protein (MCP)-1, as well as phosphorylation of c-Jun *N*-terminal kinase/stress activated protein kinase (JNK/SAPK), p38 mitogen-activated protein kinases (MAPK), and extracellular signal-regulated kinases 1/2 (ERK1/2). These data demonstrate that XB exhibits anti-diabetic, hypolipogenic, and anti-inflammatory effects via regulation of the miR-122a/33a axis in db/db mice.

## 1. Introduction

Type 2 diabetes, the most frequently occurring type, currently accounts for 85%–90% of all diabetes cases. It is characterized as a metabolic disorder involving hyperglycemia and insulin resistance [1,2]. The prevalence of diabetes has dramatically increased over the past few decades. In 2010, its prevalence was 6.4%, and it is estimated to increase to 7.7% by 2030 [3]. Insulin resistance is the primary abnormality of individuals with diabetes. The increased metabolic demand imposed on the pancreatic β cells as a result of this resistance often leads to β cell failure [4,5]. It has been hypothesized that diabetes progresses due to a sedentary lifestyle and intake of a Western-style high-fat diet [6]. 

Patients with type 2 diabetes have a greater risk of developing atherosclerotic cardiovascular disease (ASCVD) due to dyslipidemia [7]. The characteristics of diabetic dyslipidemia include low levels of high-density lipoprotein cholesterol (HDL-C) and higher levels of low-density lipoprotein cholesterol (LDL-C) and triglycerides (TGs). These lipid changes are associated with the upregulation of free fatty acid (FFA) secondary to insulin resistance. While the correction of diabetic dyslipidemia is crucial for lowering the risk for ASCVD, the evidence-based use of statin drugs does not target FFA lipotoxicity or non-alcoholic fatty liver disease (NAFLD) [8]. Hence, there remains a need for new therapeutic agents that mediate hypolipogenic and anti-inflammatory actions at the molecular level. The liver is the primary organ for the conversion of FFAs to very low-density lipoprotein (VLDL) for export. Excessive FFAs results in the accumulation of FFAs in the liver tissues [9]. Recently, NAFLD has become the most common abnormality observed in patients with diabetes. NAFLD is also strongly associated with hyperglycemia, and is a predictor of insulin resistance in adults [10,11]. 

MicroRNAs (miRNAs) are non-coding RNAs that are 20–25 nucleotides in length and serve as posttranscriptional regulators of target gene expression. To inhibit protein translation, miRNAs directly bind to complementary sequences in the 3′ untranslated region (UTR) of specific target mRNAs [12]. They have an important role in a variety of biological and disease processes by regulating several metabolic pathways [13]. In particular, miR-122a is a liver-specific miRNA that plays a pivotal role in the maintenance of a fully differentiated hepatocyte gene expression program [14] and also contributes to the β-oxidation of fatty acids (FAs) in the liver [15]. Furthermore, miR-122a is also involved in mediating oxidative stress [16], inflammation, and microsteatosis in the liver [17], which are processes known to contribute to insulin resistance in diabetes [18]. The miR-33a, a highly conserved isoform encoded in the introns of sterol response element binding protein-1 (SREBP-1), also plays an important role in the modulation of cholesterol homeostasis in the liver [19]. 

Development of type 2 diabetes is also attributed to oxidative stress and inflammation [20]. Diabetes is a condition in which the hyperglycemia and oxidation of glucose produces large amounts of free radicals, thereby leading to increased activity and expression of antioxidant enzymes [21]. Insulin resistance is also related to the production of intracellular free radicals, and strategies to downregulate intracellular reactive oxygen species (ROS) have been identified as a potential treatment for diabetes. Oxidative stress in type 2 diabetes also leads to insulin resistance by upregulating the expression of several pro-inflammatory cytokines. Adipokines, including leptin, adiponectin, tumor necrosis factor (TNF)-α, and interleukin (IL)-6, affect the development of dysfunction in lipid metabolism [22]. As a result, type 2 diabetes stimulates de novo lipogenesis, inhibits glucose uptake, and increases TG production, thereby altering the lipid metabolic parameters and leads to an increase in the levels of atherogenic cholesterol in blood [23]. 

The consumption of large amounts of sucrose is one of the risk factors for developing both obesity and related chronic diseases [24]. Fructose, a component of sugar, has been shown to induce insulin resistance and hepatic fibrosis by enhancing signaling pathways mediated by c-Jun N-terminal kinases (JNKs) and pro-inflammatory cytokines [25,26]. Consequently, numerous studies have been conducted to develop new types of sugars and sugar substitutes to obtain nutritional and beneficial effects for the management of obesity and complications arising due to diabetes. One such carbohydrate is d-allulose, a C-3 epimer of fructose, that has been reported to alter lipid-regulating enzyme activities in an obese mouse model [27]. Dietary palatinose, a sucrose analogue composed of α-1,6-linked fructose and glucose, has also exhibited significant improvements in diet-induced metabolic abnormalities, which help to prevent obesity and its complications [28,29].

Xylobiose (XB) is a β-1,4-linked d-xylose dimer and is a major component of xylo-oligosaccharides (XOS). XB is relatively abundant in bamboo and is traditionally consumed in Japan and China [30,31]. The sweetness of XB is nearly 40% less than that of sucrose and the caloric value of XOS has been confirmed as 4.0 kcal/g [32,33]. Since XB cannot be hydrolyzed by digestive enzymes present in the saliva, gastric juices, intestinal mucosa, or pancreas, it is not excreted in the urine or feces within 24 h of it being orally administered [34]. XOS has been found to reduce levels of cholesterol and blood glucose [31], revitalize the growth of intestinal bifidobacteria, activate the immune system, and mediate an anti-cancer effect [35]. In the food industry, XOS has been used as a soluble dietary fiber since it is not absorbed by the digestive systems. However, while XB is a major component of XOS, there is limited evidence regarding the possible effect of XB alone in dyslipidemia and its molecular mechanism(s) in diabetes. We hypothesized that XB exerts its anti-diabetic effects and suppresses lipogenic mechanisms in type 2 diabetic mice by regulating miR-122a/33a. Therefore, the aim of the present study was to determine whether XB is beneficial for hyperglycemia and dyslipidemia via its regulation of hepatic lipogenic genes and pro-inflammatory cytokines.

## 2. Materials and Methods

### 2.1. XB Extraction

**Figure 1:**
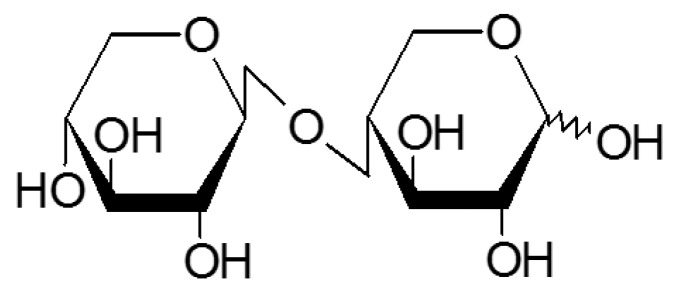
Structure of xylobiose (4-*O*-β-d-Xylopyranosyl-d-xylose).

XB was provided by TS Corporation, R&D Center (Incheon, Korea). XOS (XOS-95P, Shandong Longlive Bio-technology Co., Ltd., Qingdao, China) was diluted to 250 g/L in distilled deionized water (DDW) to make feed components that were loaded into a stimulated moving bed (SMB) as part of the process of separating XB from XOS-95P. This process was modified from a previous study [36]. Briefly, a Sugar-Pak I column (Waters, Milford, MA, USA) was used with an Agilent 1100 series high-performance liquid chromatography (HPLC) system (Agilent, Santa Clara, CA, USA) to purify XB. DDW was used for the mobile phase (0.5 mL/min) and the column oven temperature was 80 °C. A refractive index detector was used for detection. The purity of XB was 98% and the structure of XB is shown in Figure 1. 

### 2.2. Animals and Diet

Five-week-old male C57BL/6 (21–23 g) and C57BL/KsJ-db/db (17–24 g) mice were purchased from Central Lab Animal Inc. (Seoul, Korea). Mice were housed in plastic cages with standard conditions of 22 ± 2 °C, 50% ± 5% humidity, and a 12 h light–dark cycle. The mice were provided water and a modified American Institute of Nutrition (AIN)-93G diet (Unifaith Inc., Seoul, Korea). After a 14-day acclimation period, the mice were randomized into three dietary groups: (i) normal control C57BL/6 mice that received an AIN-93G diet (Ctrl, *n* = 11); (ii) diabetic control C57BL/KSJ-db/db mice that received an AIN-93G diet (DB, *n* = 10); and (iii) C57BL/KSJ-db/db mice that received an AIN-93G diet with 5% of the total sucrose content supplemented with XB (XB 5, *n* = 10). The dietary compositions are shown in Table 1; the three groups were maintained on each diet for six weeks. XB was stored in an auto-desiccator (Sanpla Dry Keeper, Sanplatec Corp., Osaka, Japan). Both body weight and food intake were monitored and recorded twice a week. All experimental protocols for this study were approved by the Institutional Animal Care and Use Committee of Ewha Womans University (IACUC 15-015).

### 2.3. Oral Glucose Tolerance Test (OGTT)

OGTTs were performed after four weeks of XB treatment. Briefly, all three groups of mice were fasted overnight, following which they were fed a glucose solution (1 g/kg bodyweight (b.w.)) by oral gavage. Blood samples were subsequently collected from the tail vein 0, 30, 60, 90, and 120 min later. Fasting blood glucose (FBG) concentrations were detected with a portable glucometer (Roche, Mannheim, Germany). OGTT area under the curve (AUC) was calculated using the trapezoidal rule [37].

### 2.4. Biochemical Analysis of Blood Samples

Plasma concentrations of TG (#AM157S-K), total cholesterol (TC, #AM202-K), HDL-C (#AM203-K), glutamic oxaloacetic transaminase (GOT, #AM103-K), and glutamic pyruvate transaminase (GPT, #AM102-K) were measured using a commercially available kit (Asan Pharmaceutical; Seoul, Korea). LDL-C levels were calculated according to the following formula [38]: LDL-C = TC − HDL-C − (TG/5).

Plasma insulin levels were measured with commercially available enzyme-linked immunosorbent assay (ELISA) kits (Crystal Chem, Downers Grove, IL, USA). To measure insulin sensitivity, homeostasis model assessment-estimated insulin resistance (HOMA-IR) was calculated: [fasting plasma insulin (μg/L) × fasting blood glucose (mg/dL)]/22.5. Commercially available kits were also used to determine the hemoglobin A1c (HbA1c) percentage (#80310, Crystal Chem) in whole blood and superoxide dismutase (SOD) activity (#706002, Cayman Chemical Company, Ann Arbor, MI, USA). All the experiments were analyzed according to the manufacturer’s instructions. 

### 2.5. Western Blotting Analysis

Liver tissues were homogenized with cold PRO-PREP protein extract solution (Intron Biotechnology, Seoul, Korea) and centrifuged at 12,000× *g* at 4 °C. The supernatants were then collected and denatured before being separated with sodium dodecyl sulfate-polyacrylamide gel electrophoresis (SDS-PAGE). The proteins were transferred to polyvinylidene difluoride membranes (PVDF) and blocked with 5% skim milk in Tris-buffered saline containing Tween-20 (TBST) buffer. Membranes were then incubated with the following primary antibodies, overnight at 4 °C: rabbit anti-glutathione peroxidase (GPX, Abfrontier, Seoul, Korea), rabbit ant-catalase (Abfrontier), mouse anti-cyclooxygenase-2 (COX-2), rabbit anti-nitric oxide synthase (iNOS) (Santa Cruz Biotechnology, Santa Cruz, CA, USA), rabbit anti-stress activated protein kinase (SAPK)/JNK, mouse anti-phospho SAPK/JNK, mouse anti-extracellular signal-regulated kinases (ERK) (1/2), rabbit anti-phospho ERK (1/2), rabbit anti-p38 mitogen-activated protein kinases (MAPK), and rabbit anti-phospho p38 MAPK (Cell Signaling, Danvers, MA, USA). After the membranes were washed, they were incubated with an appropriate secondary rabbit or mouse IgG-conjugated horseradish peroxidase antibody (Santa Cruz Biotechnology) for 1 h at room temperature. Bound antibodies were detected with an enhanced chemiluminescence (ECL) detection reagent (Animal Genetics Inc., Suwon, Kyonggi-do, Korea). Levels of α-tubulin (Sigma Aldrich, St. Louis, MO, USA) were detected as a loading control.

### 2.6. RNA Isolation and Reverse Transcriptase Polymerase Chain Reaction (RT-PCR) Analysis

Total RNA was isolated from liver tissues using TRIzol reagent (Invitrogen, Carlsbad, CA, USA), and cDNA was synthesized from each RNA sample (1 μg) by reverse transcription with a RevertAid First Strand cDNA Synthesis Kit (Fermentas, Vilnius, Lithuania). PCR amplification was performed with Taq polymerase (TAKARA, Tokyo, Japan) underthe following conditions: an initial incubation at 94 °C for 5 min was followed by denaturation at 94 °C for 30 s, annealing at 56 °C for 30 s, and an extension at 72 °C for 2 min. The resulting PCR products were separated on a 2% agarose gel containing ethidium bromide. Sequences of the primers are listed in Table 2. 

### 2.7. MicroRNA Quantification by Real-Time qRT-PCR

Total RNA was isolated and extracted from liver tissues to generate cDNA by reverse transcription with the micro Script II RT kit (Qiagen, Frederick, MD, USA). Mature miRNA levels were analyzed using the miScript primer assays (Qiagen) and miScript SYBR Green PCR kits (Qiagen), according to the manufacturer’s protocols. Amplification was performed using a Rotor-Gene Q instrument (Qiagen) with the following steps: 95 °C for 15 min and 40 cycles at 94 °C for 15 s, followed by 55 °C for 30 s, and 70 °C for 30 s. The miRNA expressions levels were normalized to the expression levels of RNA U6 and the fold change values for the miRNAs were determined according to the ΔΔ**Ct** method, as previously described [39]. 

### 2.8. Histopathological Evaluation of Liver Lesions

The left lobe of the liver tissues were fixed in 10% phosphate-buffered formalin, dehydrated in a gradient series of ethyl alcohol, and embedded in paraffin for sectioning. Sections (5 µm) were cut, and deparaffinized in xylene, rehydrated in a reverse-gradients series of ethyl alcohol, and stained with hematoxylin and eosin (HE). Two independent pathologists examined the sections under a light microscope (Olympus Co., Tokyo, Japan). 

### 2.9. Statistical Analyses

Results are represented as the mean ± standard error of the mean (SEM) for each group. Statistically significant differences between the groups were analyzed with one-way analysis of variance (ANOVA), followed by a post hoc Newman–Keuls test (GraphPad Software, San Diego, CA, USA). Data labeled with superscript letters are significantly different (*p* < 0.05). 

## 3. Results

### 3.1. Body Weight, Food and Water Intake, and Plasma Lipid Profiles

Over the course of the six-week experimental period, the body weight, food and water intake, and plasma lipid profiles were measured for all three groups of db/db/mice (Table 3). After six weeks, the DB group had a significantly higher body weight than the Ctrl group (*p* < 0.001), while XB supplementation did not affect body weight changes compared with the DB group. Regarding food and water intakes, the DB group consumed more food and water than the Ctrl group (*p* < 0.001), and the food and water intake for the XB 5 group were significantly lower than those of the DB group (*p* < 0.01 and *p* < 0.001, respectively). The lipid profiles of the DB group included significantly higher levels of TGs (*p* < 0.01), TC, HDL-C, and LDL-C as compared to the Ctrl group (*p* < 0.001 for all). In contrast, the XB 5 supplementation group had significantly lower plasma levels of TG (*p* < 0.001), TC (*p* < 0.05) and LDL-C (*p* < 0.05) compared to the DB group. The plasma HDL-C levels did not significantly differ between the DB and XB 5 groups. The levels of GOT and GPT were also significantly higher in the DB group compared to the Ctrl group (*p* < 0.001), whereas the levels in the XB 5 supplementation group did not significantly differ from the DB group.

### 3.2. OGTT, FBG, and Related Biochemical Profiles

**Figure 2:**
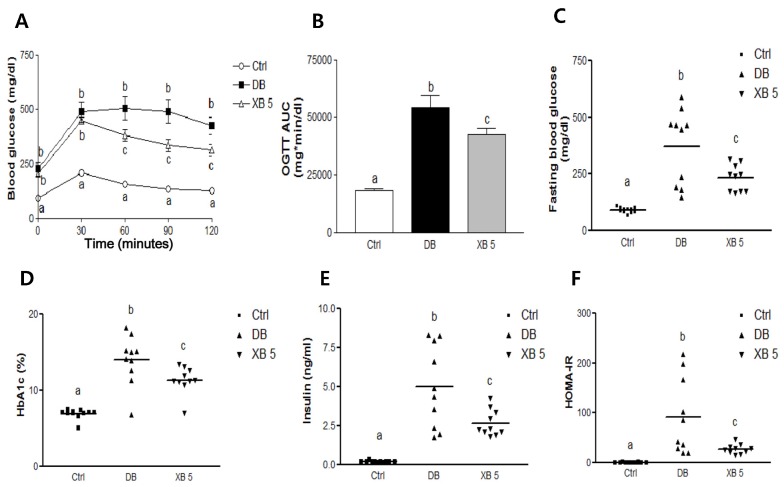
Oral glucose tolerance test (OGTT), fasting blood glucose (FBG), and blood glucose related biochemical profiles. (**A**) OGTT was measured every 30 min following administration of a glucose solution (1 g/kg b.w.) to the indicated mice; (**B**) OGTT area under the curve (AUC) was calculated. (C-F) Scatter dots represent individual mice, with bars indicating the mean of the group; (**C**) FBG; (**D**) HbA1c; and (**E**) insulin levels were measured; (**F**) HOMA-IR was calculated. Data were analyzed using one-way ANOVA with Newman–Keuls post hoc test (Ctrl, *n* = 11; DB, *n* = 10; XB 5, *n* = 10). ^a,b,c^ For a given column, data not sharing a common superscript letter significantly differ (*p* < 0.05). HbA1c, hemoglobin A1c; HOMA-IR, homeostasis model assessment-estimated insulin resistance; Ctrl, non-diabetic mice; DB, diabetic mice; XB 5, diabetic mice that received an AIN-93G diet with 5% of the total sucrose content replaced with xylobiose.

After four weeks of XB treatment, OGTTs were administered. For all three groups, the blood glucose levels peaked at the 30 min time point, after which they gradually decreased up to 120 min. However, the blood glucose levels were significantly higher in the DB group compared to the Ctrl group at the 30 min time point, and this difference was sustained for all the subsequent assay points up to 120 min (Figure 2A; *p* < 0.001). The blood glucose levels for the XB 5 group started to be significantly lower than the DB group at the 60 min time point (*p* < 0.05), which persisted through the 120 min time point (*p* < 0.05). The AUC for the OGTT data was calculated, and the DB group showed a 3-fold higher AUC compared to the Ctrl group (Figure 2B; *p* < 0.001). The OGTT AUC for the XB supplementation group was 21% lower than that for the DB group (*p* < 0.05). 

FBG, HbA1c, insulin level, and HOMA-IR were also measured for each of the three experimental groups. The DB group exhibited approximately a four-fold higher FBG level compared to the Ctrl group (*p* < 0.001), whereas the XB supplementation group exhibited a 38% lower fasting blood glucose level compared to the DB group (*p* < 0.01). Similarly, the HbA1c (*p* < 0.001), plasma insulin (*p* < 0.001), and HOMA-IR (*p* < 0.001) levels of the DB group were significantly higher than those of the Ctrl group, and these increases were significantly inhibited by XB supplementation (*p* < 0.01 for all). 

### 3.3. Hepatic Histology

**Figure 3:**
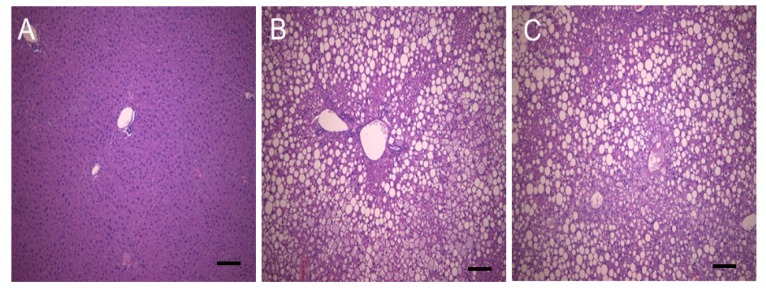
Histopathological features of mouse liver tissues. Representative tissue sections are shown for (**A**) Ctrl; (**B**) DB; and (**C**) XB 5 mice. Bar: 50 μm. Ctrl, non-diabetic mice; DB, diabetic mice; XB 5, diabetic mice that received an AIN-93G diet with 5% of the total sucrose content replaced with xylobiose.

Both the cellular and lobular structures exhibited normal morphology in the Ctrl mouse livers (Figure 3). In contrast, liver tissues from the DB group exhibited severe fatty changes with swelling of hepatic cells and inflammatory cells infiltration. The fatty changes did not markedly differ between the DB group and the XB 5 group.

### 3.4. Expression of Hepatic miRNAs Associated with Lipid Metabolism

**Figure 4:**
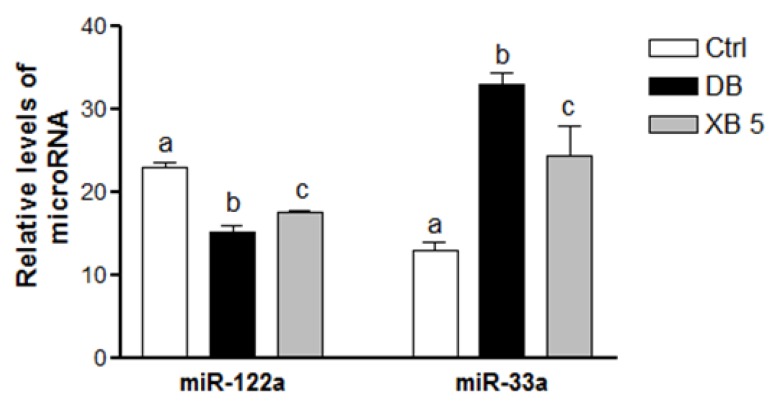
Levels of hepatic miR-122a and miR-33a in the liver. Levels of miR-122a and miR-33a detected in liver tissues were normalized to RNA U6 levels and the values shown are the mean ± SEM. ^a,b,c^ For a given column, data not sharing a common superscript letter significantly differ (*p* < 0.05; Ctrl, *n* = 11; DB, *n* = 10; XB 5, *n* = 10). Ctrl, non-diabetic mice; DB, diabetic mice; XB 5, diabetic mice that received an AIN-93G diet with 5% of the total sucrose content replaced with xylobiose.

To examine whether XB supplementation regulates diabetes-related hepatic lipid metabolism by regulating miRNAs, the levels of miR-122a and miR-33a were analyzed in liver tissues using quantitative PCR (Figure 4). Compared to the Ctrl group, a 34% decrease in miR-122a level were detected in the DB group (*p* < 0.001), but there was a 16% increase in the XB 5 group as compared to the DB group (*p* < 0.01). In contrast, the levels of miR-33a in the DB group were 61% higher (*p* < 0.001) and their levels in the XB 5 group were 36% lower than DB group (*p* < 0.05). 

### 3.5. Expression Profiles of Genes Involved in Hepatic Lipogenesis and Cholesterol Homeostasis

**Figure 5:**
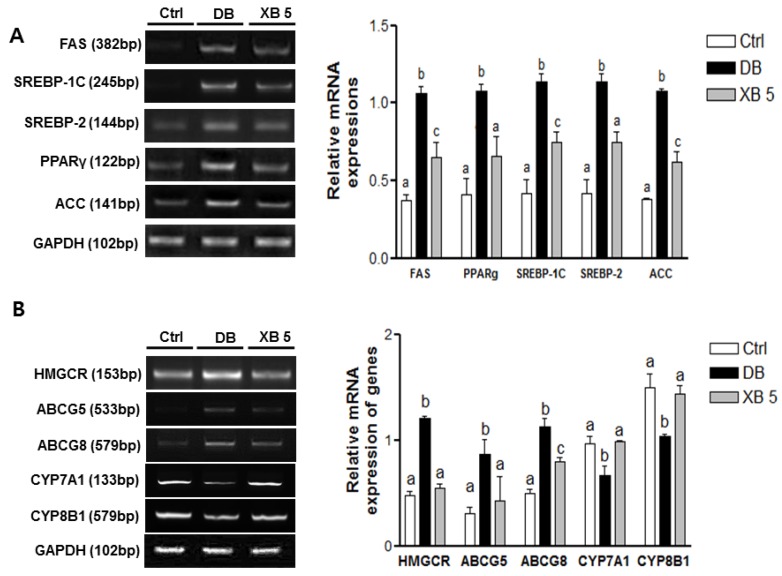
Changes in hepatic lipogenesis and cholesterol homeostasis genes. (**A**) Lipogenesis-related genes, including FAS (Fasn), PPARγ (Pparg), SREBP-1C (Srebf1), SREBP-2 (Srebf2), and ACC (Acaca) mRNA expressions were analyzed in liver tissues; (**B**) cholesterol homeostasis-related genes, including HMGCR (Hmgcr), ABCG5 (Abcg5), ABCG8 (Abcg8), CYP7A1 (Cyp7a1), and CYP8B1 (Cyp8b1) mRNA, were analyzed in liver tissues. Representative blots are shown in the left panel. Band intensities were quantified by densitometry and analyzed with one-way ANOVA with Newman–Keuls post hoc test. GAPDH (Gapdh) was used as the loading control. The values shown are the mean ± SEM. ^a,b,c^ For a given column, data not sharing a common superscript letter significantly differ (*p* < 0.05; Ctrl, *n* = 11; DB, *n* = 10; XB 5, *n* = 10). Ctrl, non-diabetic mice; DB, diabetic mice; XB 5, diabetic mice that received an AIN-93G diet with 5% of the total sucrose content replaced with xylobiose.

Gene expressions levels of fatty acid synthase (FAS, Fasn), sterol regulatory element-binding protein-1C (SREBP-1C, Srebf1), sterol regulatory element-binding protein-2 (SREBP-2, Srebf2), peroxisome proliferator-activated receptor γ (PPARγ, Pparg), and acetyl-coenzyme A carboxylase (ACC, Acaca) were analyzed to investigate the effect of XB on lipogenesis in the liver tissues (Figure 5A). We observed that the expression levels of all these lipogenesis-related genes were higher in the DB group than in the Ctrl group (*p* < 0.001 for all). However, in the XB supplementation group, more than 30% decrease in mRNA levels for these five genes were detected, as compared with the DB group (Figure 5A; *p* < 0.001 for all, respectively). 

The expression levels of genes related to cholesterol homeostasis were also examined (Figure 5B). These included: HMGCR (Hmcgr), which mediates a rate-limiting step in cholesterol biosynthesis [40], the sterol transporters, ABCG5 (Abcg5) and ABCG8 (Abcg8) [40], CYP7A1 (Cyp7a1) and sterol 12-alpha-hydroxylase (CYP8B1, Cyp8b1). The mRNA levels of HMGCR (Hmgcr), ABCG5 (Abcg5), and ABCG8 (Abcg8) were significantly upregulated in the DB group compared with the Ctrl group, and these increases were suppressed in the XB supplementation group (Figure 5B). In contrast, the mRNA levels of CYP7A1 (Cyp71a) and CYP8B1 (Cyp8b1) were lower by 31% and 30% respectively, in the DB group compared with the Ctrl group (*p* < 0.05 for both), and the levels of these genes in the XB 5 group increased by 48% and 38%, respectively, compared to the DB group (*p* < 0.05 for both).

### 3.6. Hepatic Oxidative Stress

**Figure 6:**
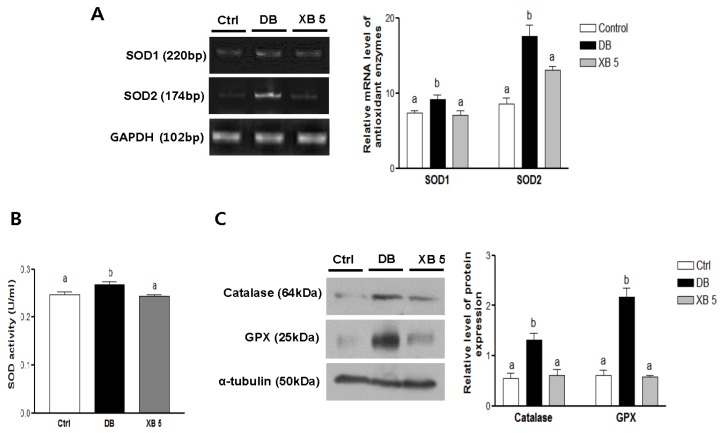
Hepatic oxidative stress-related enzymes. (**A**) Hepatic mRNA levels of SOD1 (sod1) and SOD2 (sod2) were evaluated using RT PCR. GAPDH (Gapdh) was used as the loading control; (**B**) plasma SOD activity was assessed with ELISA; (**C**) protein expressions levels of hepatic catalase and GPX in the liver tissues were detected with Western blotting. Alpha-tubulin was used as the loading control. Band intensities were quantified by densitometry and were analyzed with one-way ANOVA with Newman–Keuls post hoc test. The values shown are the mean ± SEM. ^a,b,c^ For a given column, data not sharing a common superscript letter significantly differ (*p* < 0.05; Ctrl, *n* = 11; DB, *n* = 10; XB 5, *n* = 10). Ctrl, non-diabetic mice; DB, diabetic mice; XB 5, diabetic mice that received an AIN-93G diet with 5% of the total sucrose content replaced with xylobiose.

To elucidate the regulation of hepatic antioxidant status following XB supplementation, antioxidant enzymes were analyzed in liver tissues (Figure 6). Compared with the Ctrl group, mRNA expressions levels of SOD1 (Sod1) and SOD2 (Sod2) increased by 20% and 51%, respectively, in the DB group (Figure 6A; *p* < 0.05 and *p* < 0.001, respectively). In mice administered the XB supplementation, these levels returned to the levels of the Ctrl group (*p* < 0.05 and *p* < 0.01). Similarly, SOD activity was higher in the plasma samples collected from the DB group and lower in those from the XB supplementation group, as compared to the Ctrl group (Figure 6B; each *p* < 0.001). In addition, significant increases in the levels of hepatic catalase and GPX protein expression were detected in the DB group compared with the Ctrl group (Figure 6C; each *p* < 0.001), and these increases were blocked in the XB supplementation group (*p* < 0.01 and *p* < 0.001, respectively). 

### 3.7. The Expressions of Genes Related to Inflammatory Response

**Figure 7:**
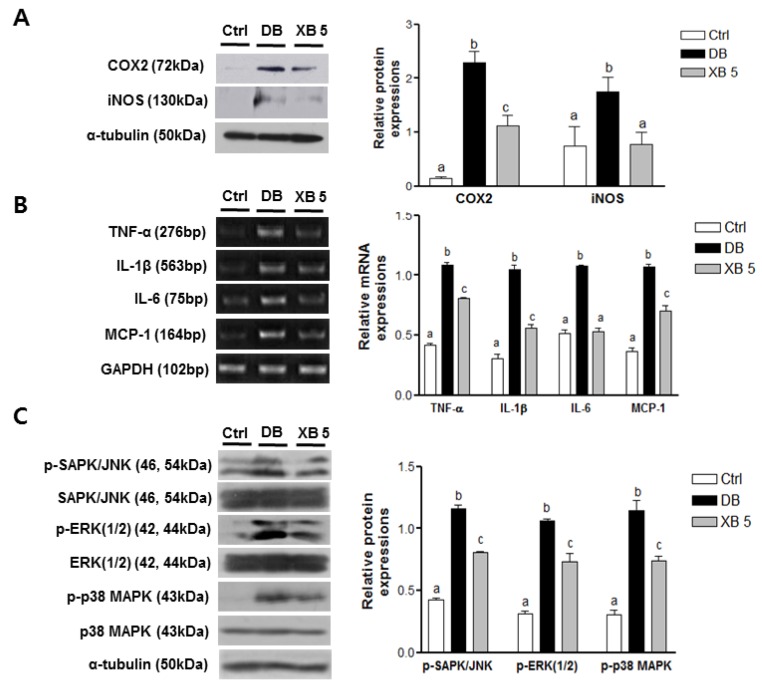
Levels of pro-inflammatory markers and phosphorylated MAPKs in the liver. (**A**) Protein levels of COX2 and iNOS were detected in liver tissues by Western blotting; (**B**) RT-PCR was used to detect mRNA levels of TNF-α (Tnf), IL-1β (Il1b), IL-6 (Il6), and MCP-1 (Mcpt1) in liver tissues; (**C**) phosphorylation of MAPKs in liver tissues were analyzed by Western blotting. Representative blots are shown in the left panels. Band intensities were quantified by densitometry (right panels) and analyzed with one-way ANOVA with Newman–Keuls post hoc test. GAPDH (Gapdh) and α-tubulin were used as loading controls. The values shown are the mean ± SEM. ^a,b,c^ For a given column, data not sharing a common superscript letter significantly differ (*p* < 0.05; Ctrl, *n* = 11; DB, *n* = 10; XB 5, *n* = 10). Ctrl, non-diabetic mice; DB, diabetic mice; XB 5, diabetic mice that received an AIN-93G diet with 5% of the total sucrose content replaced with xylobiose.

To examine the effects of XB supplementation on hepatic inflammation, expression levels of COX-2 and iNOS were analyzed by Western blotting (Figure 7A). Compared with the Ctrl group, protein levels of COX-2 and iNOS were significantly upregulated in the DB group (*p* < 0.001 and *p* < 0.05, respectively), while these levels were significantly reduced in the XB supplementation group (*p* < 0.01 and *p* < 0.05, respectively). Similarly, mRNA expression levels of the pro-inflammatory cytokines, TNF-α (Tnf), IL-1β (Il1b), IL-6 (Il6), and monocyte chemoattractant protein MCP-1 (Mcpt1) were significantly higher in the DB group by more than two-fold compared with the levels for the Ctrl group (Figure 7B; each *p* < 0.001). In contrast, XB supplementation significantly downregulated the mRNA levels of all four genes compared to the DB group (each *p* < 0.001). 

MAPKs signaling pathways are regulated by inflammatory cytokines. Therefore, the effects of XB on phosphorylation of SAPK/JNK, ERK (1/2), and p-38 MAPK were evaluated (Figure 7C). In the DB group, significantly higher phosphorylation levels were detected for each of these MAPKs (*p* < 0.001 for each). In contrast, XB supplementation suppressed phosphorylation of SAPK/JNK by 30%, ERK (1/2) by 36%, and p38 MAPK by 31% (Figure 7C; *p* < 0.01 for each).

## 4. Discussion

In the present study, intake of XB significantly suppressed the increase of plasma glucose, insulin, and blood cholesterols levels in db/db mice. Correspondingly, molecular analyses of the hepatic tissues revealed that the mice supplemented with XB exhibited reduced lipogenesis and expression of cholesterol metabolism-related genes. The expression of antioxidant enzymes and inflammatory cytokines was affected as well. Thus, the present results support the use of XB as a potential treatment for dyslipidemia and type 2 diabetes.

XB is a major component and contributor to most of the physiological activities of XOS [30]. XOS is approximately half as sweet as sucrose and has shown to have various physiologic effects in humans, such as reduction of cholesterol levels and suppression of precancerous colon lesions [41]. In a randomized double-blind clinical trial with type 2 diabetic patients in Taiwan, XOS supplementation was administered for eight weeks, and this treatment was effective in reducing the blood lipid profile and blood glucose levels [42]. XOS also exhibits hypoglycemic and hypocholesterolemic properties, and complications in kidney tissues improve by regulation of antioxidant enzymes in streptozotocin (STZ)-induced diabetic rats [43]. 

d-Xylose is a natural pentose and a potent sucrose inhibitor [44]. A recent study showed that the administration of 5% or 10% d-xylose in sucrose mixtures significantly lowered blood glucose levels in humans [45]. Furthermore, administration of 10% d-xylose in a sucrose solution significantly reduced under the glucose curve (AUCg) values and under the insulin curve (AUCi) values obtained from OGTTs performed for healthy individuals [46]. Improved lipid metabolism in serum and gene expression in adipose and liver tissues following administration of D-xylose have also been investigated in an animal model [44]. 

An increase in plasma lipids is associated with diabetes and diseases related to diabetes such as NAFLD and dyslipidemia. In the present study, XB supplementation significantly attenuated the plasma levels of TG, TC, and LDL-C, and significantly reduced FBG, OGTT AUC, insulin levels, and HOMA-IR. The OGTT and HbA1c are widely used for detection of type 2 diabetes and FBG levels are enhanced by insulin resistance that triggers complications in the liver. Both FBG and HbA1c are known predictors of type 2 diabetes and cardiovascular disease [47]. Therefore, our results indicate that XB has the capacity to mediate hypolipogenic and anti-dysglycemic effects by reducing atherogenic risk factors in blood. 

To elucidate the mechanism by which XB supplementation lowers the cholesterol levels and improves insulin resistance, two major miRNAs in lipid metabolism, hepatic miR-122a and miR-33a, were evaluated. The crucial role of miRNAs in diabetes, in particular, in regulating the pancreatic β-cell function, has recently been reported [48]. In addition, miR-122a, which is highly expressed in the liver tissue, has been found to be regulated by various lipid metabolism-related genes, including FAS, SREBP-1C, SREBP-2, and HMGCR [14]. In the present study, higher levels of miR-122a were detected in the XB group compared with DB group, and this may be the result of suppressed lipid accumulation in the liver. In STZ-induced type 1 diabetic mice, lower levels of miR-122a have been found [49], while coffee tocopherols have been found to upregulate miR-122a while also suppressing SREBP-1C, ACC, and malonyl CoA in C58BL/6J mice, concomitant with upregulation of miR-122a levels [50]. In the liver, miR-122a also has key roles in oxidative stress [16], inflammation, and microsteatosis [17], and these contribute to insulin resistance in diabetes [18]. Correspondingly, we observed a downregulation of miR-122a in the DB group, and XB supplementation ameliorated this decrease. In a study by Yang et al., lower levels of miR-122 led to insulin resistance by increasing the protein levels of tyrosine phosphatase 1B (PTP1B) [18]. Additionally, Song et al. reported that liver injury resulted in a decrease in miR-122 expression, and showed a good correlation with oxidative stress [16]. 

MiR-33a is another major regulator of cholesterol homeostasis in the liver and XB supplementation suppressed the levels of miR-33 that are otherwise elevated in db/db mice. A previous study demonstrated that when the conversion of cholesterol to bile acids was enhanced, SREBP-2 expression was induced to stimulate cholesterol synthesis, and miR-33a inhibited mRNA expression of CYP7A1, a rate-limiting enzyme in the bile acid synthesis pathway [19]. In the present study, the feed-forward activation of CYP7A1 activity by cholesterol was inhibited by miR-33a in the DB group. Furthermore, in response to increased cholesterol synthesis that occurs with diabetes, feed-forward activation of ABCG5/8 enzyme activity stimulates the conversion of cholesterol to bile acid. In our study, we observed that XB supplementation suppressed the levels of miR-33a, while increasing levels of Cyp7A1 and Cyp8B1 mRNA. Taken together, these results indicate that XB supplementation may represent a promising strategy for treating diabetes by regulating expression levels of miRNA that promotes the conversion of cholesterol to bile acid. Furthermore, based on the report that inhibition of miR-33a enhances FA oxidation and insulin signaling [51], the positive effects of XB on miR-33a may be related to improved insulin resistance. 

The liver has a vital role in maintaining glucose homeostasis, and NAFLD is closely associated with a higher accumulation of hepatic fat, insulin resistance, and type 2 diabetes [10,11]. A histological analysis of liver tissue demonstrated that XB supplementation did not markedly change the accumulation of lipid droplets, although plasma lipid profiles and the detection of hepatic lipid metabolism-related markers were affected by XB supplementation. In the liver, specific enzymes stimulate the de novo synthesis of FFAs and cholesterol, thereby increasing the outflux of these products to blood tissues and enhanced insulin resistance [52]. SREBPs play a pivotal role in the regulation of ACC and FAS at the transcriptional level. FAS is an enzyme that plays an important role in controlling body weight and dietary intake [53]. PPAR members, including PPARα, PPARγ, and PPARδ, also regulate lipid and glucose metabolism. PPARγ is presented in adipose and liver tissues and can be stimulated by SREBP-1. In the present study, significantly elevated mRNA levels of SREBP-1C, FAS, PPARγ, and ACC were detected in the DB group, and XB supplementation attenuated the expressions of these lipogenic genes. These results may be related to plasma TG levels, since lower levels of hepatic lipid synthesis can lead to limited availability of long-chain FAs, which are integral for the synthesis of hepatic TG and changes in plasma TG concentrations [54]. 

XB supplementation also significantly lowered the plasma cholesterol levels. SREBP-2 enhances the production of enzymes involved in cholesterol synthesis such as hydroxyl-methylglutryl -coenzyme A (HMG) synthase and HMGCR [55,56]. XB supplementation effectively downregulated *SREBP-2* and *HMGCR*. It is possible that XB suppresses *SREBP-2* mRNA expression via LXRα, an oxysterol receptor that induces ABCG1/5/8, and thus reduces HMGCR to produce less free cholesterol. The production of free cholesterol increases bile acid synthesis to correct the abnormal cholesterol levels. This process involves the upregulation of both ABCG5/8 and CYP7A1/B1, while ABCG5/8, CYP7A1, and CYP8B1 regulate bile acid synthesis from cholesterol [19]. Unexpectedly, mRNA levels of CYB7A1/8B1 and ABCG5/8 were found to be upregulated in the DB group. One hypothesis is that diabetes may lead to the suppression of CYP7A1/8B1 expression by nuclear receptors that control liver cholesterol regulators such as FXR, and increased LXRα leads to induced upregulation of ABCGs 5 and 8 [57,58]. It is also likely that a decrease in the conversion of cholesterol to bile acid was the result of higher cholesterol levels in the liver of the DB group compared to the Ctrl group. In a study by Sudha et al., hepatic insulin resistance was found to decrease levels of the bile acid synthetic enzyme, CYP7B1 [59]. Correspondingly, the DB group in the present study exhibited higher insulin resistance and expressed lower levels of CYP7A1 and CYP8B1, while XB supplementation significantly upregulated these enzymes. Overall, these results indicate that XB supplementation effectively lowers plasma cholesterol levels by regulating the expression of cholesterol homeostasis-related enzymes genes, leading to improved insulin resistance. 

In diabetes, both hyperglycemia and oxidation of glucose produce large amounts of free radicals, resulting in increased activity and expressions levels of antioxidant enzymes, including GPX, catalase, and SOD [21]. In the DB group, higher FBG levels were accompanied by higher expression levels of catalase, GPX, and SOD antioxidant enzymes, as well as increased SOD activity. Higher levels of the antioxidant enzymes may be associated with increases in oxidative stress. Correspondingly, XB supplementation significantly blocked expressions of the antioxidant enzymes assayed, thereby indicating that oxidative stress in the livers of the XB group was attenuated. 

Oxidative stress also leads to increased production of inflammatory cytokines, leading to insulin resistance that is associated with type 2 diabetes. Elevated levels of TNF-α in the liver are associated with the severity of diabetes [60], and IL-1β adversely affects the insulin signaling pathway and contributes to insulin resistance [61]. An increase in the circulating levels of IL-6 and MCP from various lipogenic tissues, including adipose and liver tissues, has been shown to be a predictor of type 2 diabetes progression [62]. In the present study, XB supplementation suppressed the levels of the pro-inflammatory cytokines detected, thereby suggesting that XB could have a beneficial effect on insulin resistance and hepatic lipogenesis. 

MAPKs are stimulated in response to various stimuli, including stress, ultraviolet irradiation, and inflammatory signals [63]. In particular, phosphorylation of p38 MAPK is stimulated by the pro-inflammatory cytokines, TNF-α, IL-1β, and MCP-1, while activation of the JNK signaling pathway correlated with insulin resistance and pancreatic β function [64]. In the present study, XB inhibited the phosphorylation of the three MAPKs in the liver tissues assayed. Thus, the capacity for XB supplementation to inhibit oxidative stress, to inhibit an inflammatory response, and to inactivate MAPK pathways is consistent with the reduced blood cholesterol and blood glucose levels observed in db/db mice.

There were some limitations associated with the present study. First, only a single oral concentration of XB was administered. Therefore, additional studies are needed to evaluate the concentration–response results following the administration of multiple concentrations of XB. Second, significant histological fatty changes in the liver were not detected following XB supplementation. This may be due to the low concentration of XB administered, or the insufficient duration of the present study to allow full recovery of liver histology. However, changes in molecular markers of lipogenesis and cholesterol homeostasis were detected. It remains to be determined whether a higher concentration of XB supplementation and/or a longer study duration should be included in the design of future studies. In addition, a combination of various alternative sugar substitutes may provide additive or synergistic effects on regulating diabetes-related metabolic changes. For example, it was previously reported that the selective combination of dietary carbohydrates and fatty acids attenuated the risk of developing obesity, type 2 diabetes, and metabolic syndrome [29]. Therefore, it is important for future studies to investigate the health benefits on systemic metabolic profiles that may derive from synergistic combinations of multiple nutrients.

## 5. Conclusions

**Figure 8:**
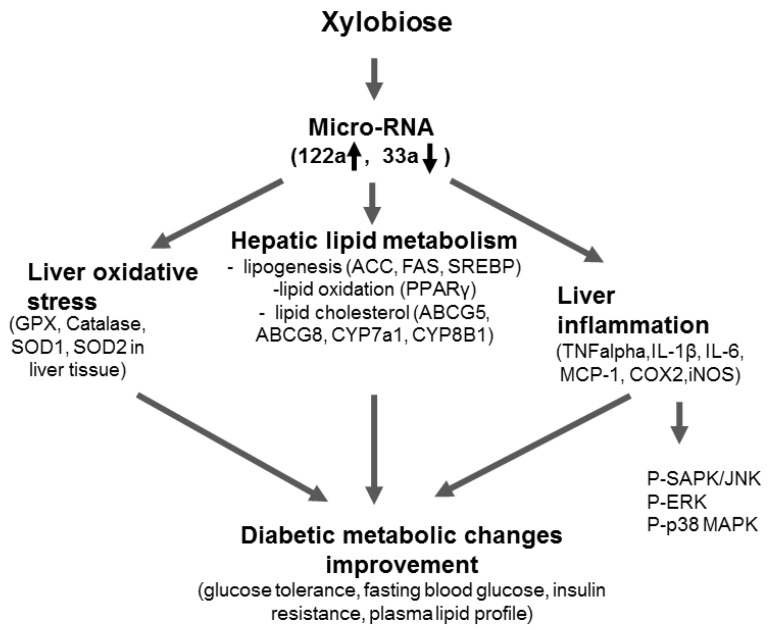
Proposed mechanism of xylobiose-regulated metabolic changes in diabetes. Xylobiose supplementation regulates the miR-122a/miR-33a axis, which in turn may regulate various lipogenesis and cholesterol homeostasis involved genes, including FAS, SREBP-1c, PPARγ, ACC, ABCG5, ABCG8, CYP7A1, and CYP8B1. In addition, xylobiose supplementation regulates oxidative stress by inhibiting the upregulation of antioxidant enzymes such as SOD1, SOD2, catalase, and GPX, while decreasing the expression of genes related to the hepatic inflammatory response by regulating phosphorylation of MAPKs.

The findings of the present study suggest that intake of XB regulates miR-122a and miR-33a in the liver, modulating the hepatic oxidative stress, lipid metabolism, and inflammation. It is possible that these modulations could prevent or reduce diabetic metabolic changes in type 2 diabetes (Figure 8). Based on the present results, novel insights into the possible application of XB as a dietary supplement for ameliorating the diabetes-related metabolic changes have been obtained, and additional clinical research studies are warranted to confirm these results and investigate the long-term health consequences.

## Figures and Tables

**Table 1:** Dietary composition for the experiment ^1^.

Ingredients (g)	AIN-93G	XB 5 ^2^
Casein, lactic	200	200
l-cystein	3	3
Corn starch	397.5	397.5
Maltodextrin	132	132
Sucrose	100	95
Xylobiose	-	5
Cellulose	50	50
Soybean Oil	70	70
Lard	-	-
Mineral mix ^3^	35	35
Dicacium phosphate	-	-
Calcium carbonate	-	-
Potassium citrate H_2_O	-	-
Vitamin mix	10	10
Cholin Bitartrate	2.5	2.5
t-butylhydroquinone	0.014	0.014
Total amount (g)	1000	1000
Total energy (kcal)	4000	4000

^1^ AIN-93G was fed to Ctrl and DB group. The experimental diet was prepared according to the AIN-93G diet with slight modifications; ^2^ Sucrose was replaced with xylobiose at 5% (XB 5) total amount of sucrose; ^3^ Mineral mixture and vitamin mixture were prepared according to AIN-93G diet. Ctrl; non-diabetic mice, DB; diabetic mice, XB 5; diabetic mice that received an AIN-93G with 5% of the total sucrose content replaced with xylobiose.

**Table 2:** The sequences of experimental primers used for RT-PCR.

	Gene Symbol	GenBank ID	Forward Primer (5′ to 3′)	Reverse primer (5′ to 3′)
FAS	Fasn	14104	CTTCGCCAACTCTACCATGG	TTCCACACCCATGAGCGAGT
PPARγ	Pparg	19016	CGAGAAGGAGAAGCTGTTGG	TCAGCGGGAAGGACTTTATGTATG
SREBP-1C	Srebf1	20787	TAGAGCATATCCCCCAGGTG	GGTACGGGCCACAAGAAGTA
SREBP-2	Srebf2	20788	CAAGAGAAAGTTCCTATCAAGCAAGTG	GTCCTTCAACTCTATGATTTTGTCGTT
HMGCR	Hmgcr	15357	TGACCTTTCTAGAGCGAGTGC	GTGCCAACTCCAATCACAAG
ACC	Acaca	107476	AGGATTTGCTGTTTCTCAGAGCTT	CAGGATCTACCCAGGCCACAT
ABCG5	Abcg5	31322257	CCTTGGTGGAACATCAAATC	TGATTTGCAGTCATGCAGTC
ABCG8	Abcg8	553727251	AGCTTCAAAGTGAGGAGTGG	AAGGACCAGGTCAAATAGCC
CYP7A1	Cyp7a1	31542444	TCAGCTCTGGAGGGAATGC	AAGTCCTCCTTAGCTGTCCG
CYP8B1	Cyp8b1	227497651	AGCTTCAAAGTGAGGAGTGG	AAGGACCAGGTCAAATAGCC
SOD1	Sod1	45597446	GAGACCTGGGCAATGTGACT	GTTTACTGCGCAATCCCAAT
SOD2	Sod2	76253932	CCGAGGAGAAGTACCACGAG	GCTTGATAGCCTCCAGCAAC
TNF-α	Tnf	21926	ATGAGCACAGAAAGCATGATC	TACAGGCTTGTCACTCGAATT
IL-1β	Il1b	16176	ATGGCAACTGTTCCTGAACTCAACT	CAGGACAGGTATAGATTCTTTCCTTT
IL-6	Il6	16193	CTCTGGGAAATCGTGGAAATG	AAGTGCATCATCGTTGTTCATACA
MCP-1	Mcpt1	17224	CCCACTCACCTGCTGCTACT	TCTGGACCCATTCCTTCTTG
GAPDH	Gapdh	14433	GCCTTCCGTGTTCCTACCC	TGCCTGCTTCACCACCTT

FAS, fatty acid synthase; PPARγ, peroxisome proliferator activated receptor γ; SREBP-1C, sterol regulatory element-binding protein-1C; SREBP-2, sterol regulatory element-binding protein-2; HMGCR, HMG-CoA reductase; ACC, acetyl-CoA carboxylase; ABCG5, ATP-binding cassette transporter G5; ABCG8, ATP-binding cassette transporter G8; CYP7A1, cholesterol 7 alpha-hydroxylase; CYP8B1, sterol 12-alpha-hydroxylase; SOD1, superoxide dismutase 1; SOD2, superoxide dismutase 2; TNF-α, tumor necrosis factor-α; IL-1β, interleukin-1β; IL-6, interleukin-6; MCP-1, monocyte chemoattractant protein-1; GAPDH, glyceraldehyde-3-phosphate dehydrogenase.

**Table 3:** The body weight, food and water intake, and plasma lipid profiles of mice fed different experimental diets for six weeks ^1^.

	Ctrl	DB	XB 5
Final body weight (g)	26.7 ± 0.5 ^a^	43.3 ± 1.1 ^b^	45.0 ± 0.6 ^b^
Food intake (g/day)	2.8 ± 0.1 ^a^	5.5 ± 0.3 ^b^	4.4 ± 0.2 ^c^
Water intake (mL/day)	4.6 ± 0.3 ^a^	18.0 ± 1.3 ^b^	11.5 ± 0.6 ^c^
Triglyceride (mg/dL)	133.0 ± 6.7 ^a^	146.5 ± 2.7 ^b^	109.4 ± 3.0 ^c^
TC (mg/dL)	107.7 ± 4.7 ^a^	163.8 ± 7.4 ^b^	149.0 ± 3.5 ^c^
HDL-Cholesterol (mg/dL)	47.1 ± 2.5 ^a^	72.7 ± 4.6 ^b^	75.5 ± 2.8 ^b^
LDL-Cholesterol (mg/dL)	34.0 ± 3.4 ^a^	61.8 ± 4.2 ^b^	51.6 ± 3.0 ^c^
GOT (Karmen/mL)	39.2 ± 20.4 ^a^	125.1 ± 31.3 ^b^	149.6 ± 25.0 ^b^
GPT (Karmen/mL)	6.4 ± 7.7 ^a^	155.7 ± 56.2 ^b^	145.6 ± 49.1 ^b^

^1^ Values shown are the mean ± standard error of the mean (SEM). Data were analyzed using one-way ANOVA and Newman–Keuls post hoc test (Ctrl, *n* = 11; DB, *n* = 10; XB 5, *n* = 10). ^a,b,c^ For a given column, data not sharing a common superscript letter significantly differ (*p* < 0.05). Ctrl; non-diabetic mice, DB; diabetic mice, XB 5; diabetic mice that received an ANI-93G diet with 5% of total sucrose content replaced with xylobiose, TC, total cholesterol; HDL, high-density lipoprotein; LDL, low-density lipoprotein; GOT, glutamic oxaloacetic transaminase; GPT, glutamic pyruvic transaminase.
